# N-Terminomics Strategies for Protease Substrates Profiling

**DOI:** 10.3390/molecules26154699

**Published:** 2021-08-03

**Authors:** Mubashir Mintoo, Amritangshu Chakravarty, Ronak Tilvawala

**Affiliations:** Department of Molecular Biosciences, University of Kansas, Lawrence, KS 66045, USA; mubashirjaved63@ku.edu (M.M.); amritangshu.chakravarty@ku.edu (A.C.)

**Keywords:** protease substrates, N-terminomics, COFRADIC, TAILS, subtiligase, CHOPS

## Abstract

Proteases play a central role in various biochemical pathways catalyzing and regulating key biological events. Proteases catalyze an irreversible post-translational modification called proteolysis by hydrolyzing peptide bonds in proteins. Given the destructive potential of proteolysis, protease activity is tightly regulated. Dysregulation of protease activity has been reported in numerous disease conditions, including cancers, neurodegenerative diseases, inflammatory conditions, cardiovascular diseases, and viral infections. The proteolytic profile of a cell, tissue, or organ is governed by protease activation, activity, and substrate specificity. Thus, identifying protease substrates and proteolytic events under physiological conditions can provide crucial information about how the change in protease regulation can alter the cellular proteolytic landscape. In recent years, mass spectrometry-based techniques called N-terminomics have become instrumental in identifying protease substrates from complex biological mixtures. N-terminomics employs the labeling and enrichment of native and neo-N-termini peptides, generated upon proteolysis followed by mass spectrometry analysis allowing protease substrate profiling directly from biological samples. In this review, we provide a brief overview of N-terminomics techniques, focusing on their strengths, weaknesses, limitations, and providing specific examples where they were successfully employed to identify protease substrates in vivo and under physiological conditions. In addition, we explore the current trends in the protease field and the potential for future developments.

## 1. Introduction

Proteases catalyze an irreversible post-translational modification called proteolysis in proteins by hydrolyzing peptide bonds [1]. More than 640 protease genes are discovered to date in the human genome, and they occupy about 3% of the entire genome [2,3]. Based on their catalytic activity, proteases are classified into six distinct classes: serine, cysteine, threonine, glutamic, aspartic, and metalloproteases [4]. Proteases are central to numerous biological pathways and play a vital role in regulating many signaling processes, such as cell-cycle progression [5], cell proliferation [6], cell death [7], DNA replication [8], tissue remodeling [9], hemostasis [10], wound healing [11], and immune responses [11]. Consistent with their essential roles, alterations in proteolytic systems result in multiple pathological conditions, including cancer [12], neurodegenerative disorders [13], infections [13], allergies [14], blood clotting disorders [15], and cardiovascular diseases [16]. Given the enormity of proteolytic potential, protease activity must be tightly regulated to prevent inappropriate and frequently destructive proteolysis. There are several mechanisms by which proteolytic activity is regulated in cells, such as regulation of gene expression [17,18], activation of their inactive zymogens [19,20,21], blockade by endogenous inhibitors [22,23], and via post-translational modifications such as glycosylation, proteolysis, and degradation [4].

Proteolytic cleavage results in the formation of two novel proteins/peptides, creating a new N- or C-terminus [24]. As a result, proteolysis changes the size, structure, and function of protein substrates. The proteolytic profile of a cell or organ is governed by protease activation, activity, and substrate specificity. Most proteases are secreted and circulated as inactive zymogens and become activated by specific cleavage upon triggering events [25]. After activation, the activity of proteases is regulated by their endogenous protein inhibitors, post-translational modifications, or pH. For example, the activity of cathepsins in the lysosomes and pepsin in gastric secretions is pH dependent [26,27]. Additionally, protease expression can be transcriptionally regulated and restricted to a specific cell type [28]. Finally, protease substrate specificity is determined based on protease-substrate interactions. Proteases cleave their substrates by recognizing either single amino acid or a particular sequence of amino acids [29,30]. Proteases with narrow specificity generally execute limited proteolysis (e.g., caspases during apoptosis), while proteases with broad specificity (e.g., cysteine cathepsins) often have significant roles in general protein degradation and clearance. Optimal protease activation, activity, and substrate specificity determine the proteome-wide signaling network of the cells, tissues, and organs—any changes or abnormalities in proteolysis result in catastrophic outcomes.

Identifying protease substrates and proteolytic events under physiological conditions can provide crucial information about how the change in protease regulation alters the entire cellular proteolytic network. However, the identification of physiological protease substrates remained challenging for a long time due to their low abundance and the unstable nature of the newly generated peptide fragments, which can be further targeted for the subsequent proteolytic degradation [31]. Additionally, the cellular heterogeneity and changes in protease expression make it challenging to dissect the relevant proteolysis from background events in complex biological mixtures [32].

In recent years, mass spectrometry-based techniques have become instrumental in identifying protease substrates under physiological conditions [32,33]. These proteomic techniques are developed by targeting the newly formed N- or C-terminal of the protease substrates and are known as N- or C-terminomics, respectively [34,35]. However, since the carboxyl group is less reactive than primary amines, the amine labeling techniques, N-terminomics, are more successfully implemented and widely used. Herein, we provide a short overview of N-terminomics techniques developed in the past decade, focusing on their strengths, weaknesses, limitations, and providing specific examples where they were successfully employed to identify protease substrates in vivo and under physiological conditions. We further explore the current trends in the protease field and the potential for future developments.

## 2. Identification of Protease Substrates Using N-Terminomics Methods

**Figure 1:**
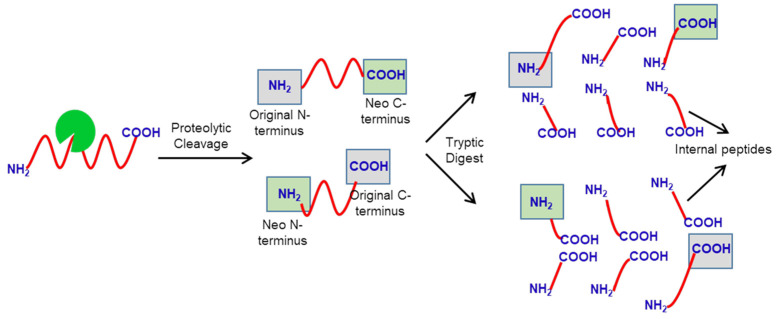
N-terminomics approach. Neo-N-terminal peptides and internal tryptic peptides. The overall workflow of the N-terminomics includes the separation of neo-N-terminal peptides from the terminal tryptic peptides.

N-terminomics methods detect the protease substrates from the complex mixture by identifying the native or neo-N-terminal generated upon proteolysis using LC-MS/MS analysis. In this review, we discuss the four major N-terminomics methods: Combinatorial Fractional Diagonal Chromatography (COFRADIC), Terminal Amine Isotopic Labeling of Substrates (TAILS), Subtiligase and Chemical enrichment of Protein Substrates (CHOPS). The most challenging step in the N-terminomics method is separating and isolating native or neo-N-terminal peptides from the internal tryptic peptides generated during the sample preparation (Figure 1). The tryptic peptides comprise more than 90% of total peptides and can easily outweigh the native or neo-N-terminal peptides in the sample. Based on how neo-N-terminal peptides are separated from the tryptic peptides, the N-terminomics methods can be further classified into negative enrichment methods and positive enrichment methods. In the negative enrichment methods (e.g., COFRADIC and TAILS), the tryptic peptides are enriched to isolate the native or neo-N-terminal peptides from the sample. While in the positive enrichment methods (e.g., Subtiligase, and CHOPS), native or neo-N-terminal peptides are directly enriched and isolated from the sample.

**Figure 2:**
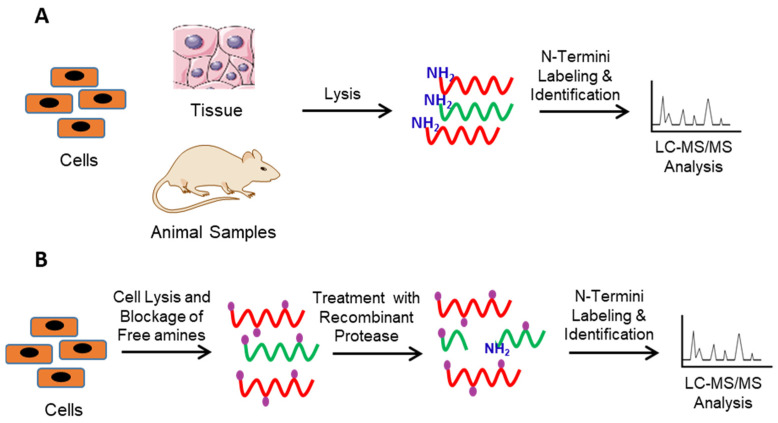
*Forward* and *Reverse* approaches of N-terminomics. (**A**) In the *forward* N-terminomics approach, treated and control samples lysed and subjected N-terminomics workflow followed by LC-MS/MS analysis to identify the global proteolysis. (**B**) In the *reverse* N-terminomics, samples are lysed, followed by the addition of protease of interest. The treated and control samples are then subjected to LC-MS/MS analysis to identify the physiological substrates and substrate specificity of the protease of interest.

N-terminomics methods can be further divided into *forward* N-terminomics and *reverse* N-terminomics [36]. The *forward* N-teminomics is used to identify the global proteolytic events, while the *reverse* N-terminomics is used to identify the proteolytic events occurring from a protease of interest (Figure 2). In the *forward* N-terminomics method, control and diseased/treated samples are lysed and subjected to N-terminomics workflow followed by LC-MS/MS analysis (Figure 2A). The identified peptides between two samples are then compared to dissect the differential proteolytic events associated with the treated/diseased samples. For example, COFRADIC, TAILS, and Subtiligase have been used to study Matrix Metallo Proteases (MMP) activity in cancer cells [37,38], cathepsin function of protein degradation, and proteolysis in pancreatic tumors and human blood samples [39]. The detailed list is compiled in Table 1. While the *forward* N-terminomics provides information about the relative proteolysis between two samples, it cannot identify specific protease substrates associated with the protease of interest. For this purpose, *reverse* N-terminomics methods are used. In this method, samples are lysed, the endogenous proteases are quenched, and native and neo-N-terminal peptides are blocked. Next, the samples are treated with the protease of interest. The control and treated samples are then subjected to N-terminomics workflow followed by LC-MS/MS analysis. Finally, the identified peptides from both samples are compared to dissect the physiological substrates of protease of interest (Figure 2B). The *reverse* N-terminomics has been successfully employed to identify physiological substrates of various proteases, including ADAMTS7 [40] and MMPs [41], using TAILS, granzyme tryptases, and several caspases using COFRADIC and Subtiligase [42,43]. The detailed list is compiled in Table 1. The *reverse* N-terminomics approach identifies physiological substrates of the target protease directly from the biological sample. Importantly, it also reveals the protease primary sequence specificity. However, *reverse* N-terminomics does not provide the functional or biological significance of the proteolytic events identified and can lead to the identification of substrates from organelles that would not normally co-localize with the protease in a living cell.

**Figure 3:**
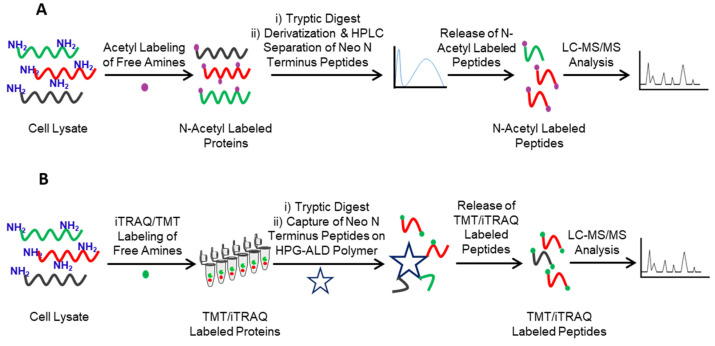
Negative enrichment approaches of N-terminomics. (**A**) In COFRADIC, the native or neo-N-terminal peptides are blocked by acetylation followed by tryptic digest. The internal tryptic peptides are then labeled and separated by HPLC, leaving acetyl-labeled native or neo-N-terminal peptides. LC-MS/MS analysis of acetyl-labeled native and neo-N-terminal peptides provide identity of protease substrates and protease cleavage sites. (**B**) In TAILS, the native or neo-N-terminal peptides are blocked with isobaric labeling followed by trypsin digestion. The internal tryptic peptides are then separated by capturing HPG-ALD polymer, leaving isobaric tag labeled native and neo-N-terminal peptides. LC-MS/MS analysis of probe-labeled native and neo-N-terminal peptides provide identity of protease substrates and protease cleavage sites.

**COFRADIC** Combinatorial Fractional Diagonal Chromatography (COFRADIC) is one of the earlier N-terminomics methods [77]. This negative N-terminomics method uses different N-termini labeling to induce chromatographic shifts to isolate the tryptic peptides from the native or neo-N-terminal peptides. In this method, the native or neo-N-terminal peptides are blocked by acetylation followed by trypsin digestion. The samples are then separated via high-performance liquid chromatography (HPLC) fractionation, and the fractions are treated with 2,4,6-trinitrobenzenesulfonic acid (TNBS). The TNBS reacts with the free amine of tryptic peptides, adding a trinitrophenyl group to tryptic peptides and increasing their hydrophobicity. Finally, the acetyl-labeled native and neo-N-terminal peptides and TNBS labeled tryptic peptides mixture are separated by the secondary HPLC run. Since TNBS labeling alters the hydrophobicity of tryptic peptides, they run at a different retention time during the secondary HPLC run, while the retention time of acetyl-labeled native and neo-N-terminal peptides remains the same during primary and secondary HPLC runs, enabling separation of tryptic peptides. These native and neo-N-terminal peptides are then collected and analyzed using LC-MS/MS analysis [78]. LC-MS/MS analysis of acetyl-labeled native or neo-N-terminal peptides provide the identity of protease substrates and the protease cleavage sites (Figure 3A). Despite having many advantages, COFRADIC also has certain limitations. Apart from being an expensive technique, sample preparation and data analysis is labor-intensive and require significant expertise. In addition, since the method includes multiple chromatographic runs, it requires a large number of samples [79].

Zahedi and co-workers recently modified COFRADIC to charge-based fractional diagonal chromatography (ChaFRADIC) [80,81]. Instead of using the modified hydrophobicity of tryptic peptides for chromatographic separation, this method uses the altered charged state of tryptic peptides to separate them from native or neo-N-N-terminal peptides. In this method, native or neo-N-terminal peptides are blocked by carbamidomethylation followed by trypsin digestion. The samples are then separated by a strong cation exchange (SCX) chromatography at pH 2.7 in different fractions, and the tryptic peptides in these fractions are blocked by acetylation, altering the charge of the tryptic peptides [82]. Finally, the carbamidomethyl-labeled native and neo-N-terminal peptides and acetyl-labeled tryptic peptides mixture are separated by the secondary SCX run. Since acetylation alters the charge of tryptic peptides, they run at a different retention time during the secondary SCX run, while the retention time of carbamidomethylated native and neo-N-terminal peptides remains the same during primary and secondary SCX runs, enabling separation of tryptic peptides. These cabamedomethyl-labeled native and neo-N-terminal peptides are then collected and analyzed using LC-MS/MS analysis [81]. LC-MS/MS analysis of native or neo-N-terminal peptides provides the identity of protease substrates and the protease cleavage sites (Figure 3A). The main advantages of ChaFRADIC are reductions in both the amount of material required and LC-MS/MS instrument time.

The COFRADIC method is widely used to study the backbone N-terminal acetylation in eukaryotes [32]. COFRADIC revealed that about 84% of human proteins and 57% of yeast proteins were acetylated at the N-terminus. The authors further noted that about 50% of the acetylation occurred on a protein containing a Met-Lys dipeptide sequence in humans, a phenomenon not observed in the yeast [44]. COFRADIC has also been used to construct the substrate degradome of apoptosis by identifying caspase substrates in human Jurkat T-lymphocytes. Using COFRADIC, the authors identified 14 different caspase substrates involved in the spliceosome, suggesting the importance of alternative splicing in apoptosis [45,83]. Further studies revealed that the initiator caspase 2 and executioner caspases 3 and 7 share a common consensus sequence DEVD|G [46]. Additionally, Impens et al. used the COFRADIC method to identify human immunodeficiency virus-1 (HIV-1) protease substrates, showing the prominent role of HIV proteases in HIV infection [47].

**TAILS** Terminal Amine Isotopic Labeling of Substrates (TAILS) an alternative negative enrichment method developed by Christopher Overall and co-workers to overcome the limitations of COFRADIC [84]. In contrast to COFRADIC, in TAILS, the internal tryptic peptides are separated using a primary amine-reactive polymer which considerably reduces the time and effort required for the separation (Figure 3B). The basic TAILS protocol starts with blocking the native and neo-N-terminal peptides using isotopic reagents such as Tandem Mass Tagging (TMT) or iTRAQ, followed by trypsin digestion. The internal tryptic peptides are then captured using high molecular weight dendritic polyglycerol aldehyde polymer (HPG-ALD). The HPG-ALD polymer covalently reacts with the amino terminus of tryptic peptides, leaving the isotopically labeled native and neo-N-terminal peptides unbound which are then recovered by ultrafiltration and analyzed using LC-MS/MS analysis. LC-MS/MS analysis of isobaric tag labeled native or neo-N-terminal peptides provide identity protease substrates and the protease cleavage sites (Figure 3B) [85]. Alternatively, Mommen et al. developed a phospho tagging (PTAG) method that can also be used to label the internal tryptic peptides, which are then depleted using titanium dioxide (TiO2), leaving the isotopically labeled native and neo-N-termini unbound, which are collected and analyzed using LC-MS/MS analysis. LC-MS/MS analysis of isobaric tag labeled native and neo-N-terminal peptides provide identification of protease substrates and the protease cleavage sites [86].

TAILS possesses several advantages over the COFRADIC approach, such as N-terminal specificity biases are minimal with this approach, a smaller quantity of starting material is required, and kits and reagents are available commercially [79]. However, despite having many advantages, TAILS also has some bottlenecks. For example, TAILS requires significant statistical analysis to distinguish the tagged and untagged N-termini in complex peptide mixtures, and the reagents used in this method are very expensive. Furthermore, the negative enrichment methods, COFRADIC and TAILS, could suffer from high false positives due to the incomplete blocking and isolation of the internal tryptic peptides [87].

Since its development, the TAILS method has been successfully applied to identify protease substrates from many different systems. Using TAILS, Klein et al. identified HOIL1 as a substrate of the paracaspase, mucosa-associated lymphoid tissue lymphoma translocation protein 1 (MALT1). In the presence of antigens, HOLT1 amplifies the immune response during the NF-kB mediated signaling [67]. Using TAILS, Gordon et al. compared the relative proteolysis between the ulcerative colitis patients and healthy controls. The authors noted that the host proteases were the major contributors to overall proteolysis. These studies further showed that proteolysis of proteins involved in neutrophil degranulation, metabolism, and adherens junction was altered in ulcerative colitis patients [65]. Additionally, TAILS has been used to identify the proteolytic profile of chronic obstructive pulmonary disease (COPD) samples. These studies identified a total of 299 human sputum proteins, out of which 125 were substrates of proteases [66].

TAILS has been widely used to identify the novel physiological substrates of metallo and other proteases. Using iTRAQ-TAILS, Padova et al. identified the substrate profile of substrates of MMP-2 and MMP-9. These studies confirmed that insulin-like growth factor-binding protein-4, thrombospondin-2, and galectin-1 are substrates of MMP-2, and MMP-9, establishing the role of their proteases in angiogenesis [41]. Moreover, Starr et al. identified the substrate profile of a neutrophil-specific membrane-type 6 matrix metalloproteinase (MTP6-MMP), which plays a role in cancer and multiple sclerosis. These studies established vimentin, cystatin C, secreted protein, acidic and rich in cysteine (SPARC), and galectin-1 as substrates of MTP6-MMP [71]. Bellac et al. used TAILS to identify the substrate profile of a macrophage-specific metalloproteinase MMP12 involved in inflammation. Authors showed that MMP-12 activates prothrombin and reduces inflammation by decreasing complement activation [72]. TAILS was also used to identify the substrate profile of metalloprotease meprin β. These studies confirm that meprin β cleaves the amyloid precursor protein, a critically important protein in neurodegeneration [73]. Moreover, Alcaraz et al. used TAILS to identify the substrates of a tumor-specific protease, cathepsin D, from triple-negative breast cancer cells. The authors reported that Cathepsin D cleaves the matricellular SPARC protein under acidic pH, generating a 9-KDa fragment, which is more oncogenic than intact SPARC [64]. TAILS was also used to identify substrates of the 3C proteinases (3C^pro^) of coxsackievirus B3 and poliovirus. These studies showed that the heterogeneous nuclear ribonucleoprotein M (hnRNP M), an RNA-binding protein that plays a role in pre-mRNA splicing, is a substrate of 3C^pro^ [75].

**Figure 4:**
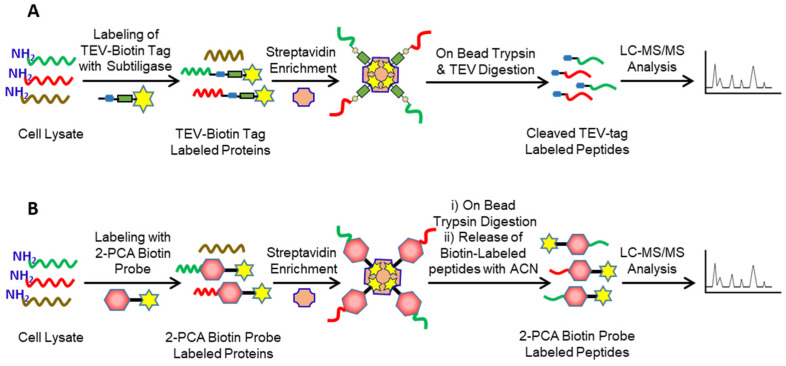
Positive enrichment approaches of N-terminomics. (**A**) In Subtiligase approach, the native or neo-N-terminal peptides labeled with the TEV-biotin probe using Subtiligase enzyme. The labeled peptides are then enriched on avidin beads followed by sequential on-bead trypsin and TEV digestion. LC-MS/MS analysis of probe-labeled native and neo-N-terminal peptides provides the identity of protease substrates and protease cleavage sites. (**B**) In CHOPS, the native or neo-N-terminal peptides are labeled with the 2PCA-biotin tag. The probe-labeled peptides are then enriched on avidin beads followed by on-bead trypsin digestion. After washing, the probe-labeled native and neo-N-terminal peptides are eluted from the beads using acetonitrile. LC-MS/MS analysis of probe-labeled native and neo-N-terminal peptides provides the identity of protease substrates and protease cleavage sites.

**Subtiligase:** In contrast to COFRADIC and TAILS, Subtiligase is a positive N-terminomics method that directly enriches and isolates native or neo-N-terminal peptides. The Subtiligase method is developed by Jim Wells and co-workers, which uses the enzymatic biotinylation of the native or neo-N-terminal peptides using a modified Subtiligase enzyme(Figure 4A) [88]. The labeled peptides are then enriched, isolated, and identified using LC-MS/MS analysis. Subtiligase is engineered from a nonspecific protease called subtilisin to selectively modify N-terminal amines while leaving the protein ε-lysine side chains unmodified. In the Subtiligase method, the native or neo-N-terminal proteins/peptides are labeled with the TEV-biotin tag using subtiligase. The probe-labeled native or neo-N-terminal peptides are then enriched on streptavidin beads, followed by sequential on-bead trypsin and Tobacco Etch Virus (TEV) digestion and elution of the probe-labeled native and neo-N-terminal peptides. The TEV-cleaved neo-N-terminal peptides consist of an α-aminobutyric acid signature, differentiating them from tryptic peptides [32]. LC-MS/MS analysis of the probe-labeled native or neo-N-terminal peptides provides identity of protease substrates and protease cleavage sites (Figure 4A). Recently, Jim Wells and co-workers further optimized the Subtiligase method to identify protease cleavage sites from the membrane proteins present on the surface of living cells, making it the only N-terminomics method that can be used to identify protease substrates from the living cells [56].

The Subtiligase approach holds many advantages over the other N-terminomics methods, such as live-cell labeling, direct enrichment, and explicit identification of neo-N-termini without ambiguity. However, despite many advantages, there are a few drawbacks, such as the starting large quantities of starting material is required for adequate enrichment, and the reagents used in this approach are not commercially available. In addition, although Subtiligase has a higher and broader specificity than other ligases, it still has biases towards specific N-terminal sequences.

Subtiligase has been widely adopted to identify the global proteolytic events and targeted protease substrate specificity from biological samples using *forward* and *reverse* N-terminomics approaches. The *forward* Substiligase N-terminomics was used to identify the proteolytic profile of human blood and plasma samples. In this study, the authors identified multiple N-terminal peptides originating from complement and coagulation cascades. Additionally, the *forward* Subtiligase was used to identify the global proteomic profile of thyroid carcinoma, colorectal cancer, Alzheimer’s disease, and cardiovascular diseases [57,59,60,61,62,63]. Overall, the Substiligase method enabled the simplification of human blood proteomes by specifically enriching the N-terminal peptides and discovering disease-specific biomarkers.

The *reverse* Substiligase N-terminomics has explicitly been used to study the substrate specificity of caspases, a class of cysteine proteases involved in apoptosis and pyroptosis, including caspases 3, 6, 7 (executioner caspases) [53,54,89], and caspases 1, 4, and 5 (inflammatory caspase) [48,49,90]. Caspases cleave their substrates predominantly after aspartate residues [50,51]. Using the *reverse* Subtiligase approach, Scaman et al. showed that apart from aspartic acid, caspases 3 and 7 can cleave after glutamic acid residues, while caspase 3 can also cleave specifically after phosphorylated serine residues [52]. In addition, using Subtiligase, Agard et al. identified about 82 caspase-1 and three caspase-4 substrates from THP-1 monocytic cell lysates. The authors further showed that caspase-1 contains a higher number of cellular substrates than caspases-4 and 5 [90]. Additionally, using the *reverse* Subtilgase approach, Hill et al. identified the substrate profile of Zika virus protease NS2B-NS3 from HEK293 cells. The authors showed that the Zika virus protease cleaves host eukaryotic translation initiation factor 4 gamma 1 (eIF4G1) and autophagy-related protein 16-1 (ATG16L1) [55].

**CHOPS:** Chemical enrichment of Protease Substrates (CHOPS) is a positive enrichment method recently developed by Daniel Bachovchin and co-workers as an alternative approach to Subtiligase [76]. In contrast to Subtiligase, CHOPS uses the chemical biotinylation of the native or neo-N-terminal peptides using a 2-pyridinecarboxaldehyde (2PCA)-biotin probe. The probe-labeled peptides are then enriched, isolated, and identified using LC-MS/MS analysis (Figure 4B). 2PCA selectively modifies N-terminal amines while leaving the protein ε-lysine side chains unmodified. In the CHOPS method, the native and neo-N-terminal proteins/peptides are labeled with the 2PCA-biotin tag. The probe-labeled are then enriched on streptavidin beads, followed by on-bead trypsin digestion. After removing tryptic peptides, the probe-labeled native and neo-N-terminal peptides are eluted from the beads using acetonitrile followed by LC-MS/MS analysis. LC-MS/MS analysis of probe-labeled native and neo-N-terminal peptides provides the identity of protease substrates and the protease cleavage sites (Figure 4B).

Since CHOPS uses chemical biotinylation instead of enzymatic biotinylation, it removed the major flaw of the Subtiligase method, i.e., biases in labeling towards N-terminal sequences, while still retaining all advantages of the Subtiligase method, including direct enrichment and explicit identification of neo-N-termini without ambiguity. However, this method still needs to be optimized for live-cell labeling. Nonetheless, CHOPS remains the most versatile and promising N-terminomics method for future use.

CHOPS was used to identify substrate specificity of dipeptidyl peptidases (DPP). The dipeptidyl peptidases (DPPs) cleave after proline residues present on the second position in polypeptide chains, releasing dipeptides from the N-termini. The DPPs regulate the activities of many biologically important molecules such as neuropeptides, cytokines, and hormones [91]. However, the identification of the DPP substrate remains challenging predominantly due to the similar size of the intact and cleaved substrates [92,93]. Griswold et al. successfully employed the CHOPS method to identify the substrate specificity of DPP8 and DPP9. The authors showed that DPP8 and DPP9 do not cleave full-length globular proteins Nlrp1b and SMAC but efficiently cleave short peptides, confirming that DPP8 and DPP9 predominately prefer small peptide substrates over full length proteins [76].

## 3. Conclusions and Future Directions

Identifying protease substrates and proteolytic events under physiological conditions is a fundamental step towards understanding the proteolytic landscape of biological samples. The N-terminomics methods discussed in this review are emerging as indispensable tools to identify and understand the global proteolytic events and protease substrate specificity under physiological conditions. The main steps in N-terminomics methods are the labeling and enrichment of the native N-termini or neo-N termini peptides generated from the proteolytic cleavage, reducing the complexity of the samples, and enabling the unambiguous identification of the protease substrates and protease cleavage sites.

In recent years, multiple positive and negative enrichment N-terminomics strategies have been developed. Although the N-terminomics field is rapidly expanding, few challenges are associated with these techniques, requiring further technological and computational progression. For example, the precise annotation of the protease cleavage sites still remains challenging as they are annotated based on protein backtracking from the single peptide hit. In addition, some unfavorably cleaved neo-N-terminal peptides cannot be detected during LC-MS/MS analysis. Furthermore, the regulation of proteases by post-translational modifications remains another challenging avenue to study by N-terminomics as these post-translational modifications can lead to a generation of non-tryptic or semi-tryptic peptides and can be difficult to detect. Such challenges can be overcome by an integrated approach, including both neo-N-terminal and neo-C-terminal peptides analysis. The simultaneous analysis of N- and C-terminomics could improve the annotation of cleavage sites and provide much more information about the global proteolytic events in complex biological mixtures.

The current N-terminomics strategies can be further used in several different directions to increase our knowledge of the complex interactions between proteases, their substrates, and inhibitors. For example, N-terminomics can be used to identify and annotate the unpredicted and undefined proteins and proteoforms to improve the existing genomic databases. Moreover, the *reverse* N-terminomics can be increasingly used to map the protein–protease interactome of the target proteases under physiological conditions. Such data can then be used to dissect the protease regulation in a proteolytic cascade and generate a proteolytic network in health and diseases. In addition, such data can help to develop a library of proteolytic biomarkers that could be used to diagnose multiple pathological conditions.

In conclusion, N-terminomics methods have opened a new horizon to study protease regulation from the complex biological mixtures, far from our reach a decade ago. More studies are underway using these techniques to decode the biological protease pathways and understand the crosstalk between proteolysis and post-translational modifications. Such studies will generate a future roadmap for identifying novel disease mechanisms, drug targets, and biomarkers for multiple pathologies.

## Figures and Tables

**Table 1:** Applications of N-terminomics methods.

Method	Applications
COFRADIC	Identification of the N-terminal acetylation sites in human and yeast proteins [44] Identifying caspase cleavage sites during apoptosis [45] Sequence specificity of initiator and executioner caspases [46] Substrate identification for HIV-1 protease [47]
Subtiligase	Identification of caspase substrates [48,49,50,51,52,53,54] Identification of the Zika virus NS2B-NS3 protease substrates [55] Protease cleavage sites on membranes and the surface of living cells [56] Identification of unique proteins and peptides in the human serum and plasma [57] and discovery of disease-specific biomarkers [57,58,59,60,61,62,63]
TAILS	Identification cathepsin D substrate profile from triple-negative breast cancer background [64] Identification of proteolytic events from pancreatic tumors [39] Identification of proteolytic events from ulcerative colitis [65] Identification of proteolytic events from COPD [66] Identification of MALT1 substrates in NF-κB mediated signaling in the presence of antigens [67] Substrate profiling of DPP8 and DPP9 proteases [68] Substrate profiling of metalloproteases [41,69,70,71,72,73] Identification of proteolytic events and protease cleavage sites during Enterococcal *E coli* [74] and viral infections [75]
CHOPS	Substrate profiling of DPP8 and DPP9 proteases [76]

## Abbreviations

Combinatorial Fractional Diagonal Chromatography (COFRADIC), Terminal Amine Isotopic Labeling of Substrates (TAILS), Chemical enrichment of Protease Substrates (CHOPS), Tobacco Etch Virus protease (TEV protease), dipeptidyl peptidases (DPP), Matrix Metallo Protease (MMP), polyglycerol aldehyde polymer (HPG-ALD), Tandem Mass Tagging (TMT), strong cation exchange chromatography (SCX).
